# Prediction of Genetic Interactions Using Machine Learning and Network Properties

**DOI:** 10.3389/fbioe.2015.00172

**Published:** 2015-10-26

**Authors:** Neel S. Madhukar, Olivier Elemento, Gaurav Pandey

**Affiliations:** ^1^Department of Physiology and Biophysics, Meyer Cancer Center, Institute for Precision Medicine and Institute for Computational Biomedicine, Weill Cornell Medical College, New York, NY, USA; ^2^Tri-Institutional Training Program in Computational Biology and Medicine, New York, NY, USA; ^3^Department of Genetics and Genomic Sciences and Graduate School of Biomedical Sciences, Icahn Institute for Genomics and Multiscale Biology, Icahn School of Medicine at Mount Sinai, New York, NY, USA

**Keywords:** genetic interactions, machine learning, prediction, cancer, drug discovery, network analysis

## Abstract

A genetic interaction (GI) is a type of interaction where the effect of one gene is modified by the effect of one or several other genes. These interactions are important for delineating functional relationships among genes and their corresponding proteins, as well as elucidating complex biological processes and diseases. An important type of GI – synthetic sickness or synthetic lethality – involves two or more genes, where the loss of either gene alone has little impact on cell viability, but the combined loss of all genes leads to a severe decrease in fitness (sickness) or cell death (lethality). The identification of GIs is an important problem for it can help delineate pathways, protein complexes, and regulatory dependencies. Synthetic lethal interactions have important clinical and biological significance, such as providing therapeutically exploitable weaknesses in tumors. While near systematic high-content screening for GIs is possible in single cell organisms such as yeast, the systematic discovery of GIs is extremely difficult in mammalian cells. Therefore, there is a great need for computational approaches to reliably predict GIs, including synthetic lethal interactions, in these organisms. Here, we review the state-of-the-art approaches, strategies, and rigorous evaluation methods for learning and predicting GIs, both under general (healthy/standard laboratory) conditions and under specific contexts, such as diseases.

## Introduction

Genetic interactions (GIs) occur when the combined phenotypic effect of mutations in two or more genes is significantly different from that expected if the effects of each individual mutation were independent of the others (Mani et al., 2008; Boucher and Jenna, 2013). They can be broadly classified as either positive or negative based on whether the double mutation leads to either a greater increase (positive) or decrease (negative) in fitness than what would be expected in the case of any individual mutation (Jasnos and Korona, 2007). These interactions are important for delineating functional relationships among genes and their corresponding proteins, as well as elucidating complex biological processes and diseases (Boucher and Jenna, 2013). A variety of GIs have been described (Mani et al., 2008), but one of the most well-studied types is synthetic lethality, which is the extreme case of negative GIs. First coined in *Drosophila* (Bridges, 1922), synthetic lethality describes how combinations of mutations confer lethality while individual ones do not. Perhaps the simplest form of synthetic lethality lies in paralogous gene relationships. Paralogous genes, especially recently duplicated ones, are frequently functionally redundant; therefore, loss of all or several of them is often necessary to obtain a phenotype (VanderSluis et al., 2010).

One of the most exciting biomedical applications of GIs lies in how synthetic lethality can be used to selectively target cancer cells (Kaelin, 2005; McLornan et al., 2014). This is exemplified by the success of pharmacological PARP inhibition in BRCA-mutated (and deficient) tumors (Weil and Chen, 2011; Food and Drug Administration, 2012). Therapeutic opportunities arising as a result of synthetic lethality may extend beyond BRCA-mutated tumors. For example, many, if not most, inactivating somatic mutations in cancer genes cannot be targeted directly, but some of them may be actionable via their synthetic lethal interactions. This may be the case for TP53, a commonly lost tumor suppressor in cancer and for which actionable synthetic lethal interactions have been identified (Emerling et al., 2013). A recent paper identified a synthetic lethal interaction between EZH2 and ARID1A in ARID1A-mutated cancers, thus providing therapeutic opportunities since EZH2 can be pharmacologically targeted (Bitler et al., 2015). While a few synthetic lethal interactions have been uncovered, the widespread loss of genomic material in most tumors likely creates even more, possibly many of which are tumor specific. This is an attractive opportunity, because targeting a gene that is synthetic lethal to a cancer-associated mutation may preferentially kill cancer cells and spare normal cells. As a proof-of-principle, a search for passenger mutations uncovered an interaction between ENO1 (lost) and enolase 2 (ENO2), and targeting the remaining enolase led to cell death (Muller et al., 2012).

A distinct but related form of GI is synthetic dosage lethality (SDL). In SDL, over-expression of one gene combined with loss of the other gene leads to cell death. SDL interactions are important in cancer because many oncogenic events are due to gene over-expression or mutations that constitutively activate a pathway in a way that mimics over-expression. This is not only the case for oncogenes such as MYC (frequently over-expressed) but also mutations such as KRAS, neither of which can be directly targeted pharmacologically. Several studies have described how SDL interactions involving these genes can be therapeutically exploited (Chan and Giaccia, 2011). For example, MYC-driven tumors are dependent on 4EBP1 [eukaryotic translation initiation factor 4E (eIF4E) binding protein 1], a mammalian target of rapamycin (mTOR) substrate. Thus, pharmacologic targeting of mTOR in MYC-driven tumors may represent a promising direction (Pourdehnad et al., 2013).

In organisms such as yeast, systematic GIs screens have been performed and have revealed hundreds of thousands of interactions (Dixon et al., 2009a,b). Such screens have also been performed in higher eukaryotes and human cell lines (Barbie et al., 2009; Luo et al., 2009; Scholl et al., 2009; Hajeri and Amatruda, 2012; Kranz and Boutros, 2014; Maia et al., 2015), albeit on a more limited scale. As described in the next section, screening techniques, such as E-MAP and SGA, are not easily applicable to higher eukaryotes. Moreover, the latter have more genes (humans have four to six times more genes than yeast) and a correspondingly larger number of gene pairs (and potentially higher combinations) to assay. As a result, relatively few GIs are known in human, murine, and other higher eukaryotic cells.

Owing to the general lack of experimentally verified GIs despite their biomedical utility, several computational approaches have been developed to identify/predict them. Some approaches have been developed for general, context-independent applications, whereas others are more directly related to specific contexts/diseases, most commonly cancer. These approaches adopt concepts from diverse areas such as machine learning, evolutionary genomics, feature engineering, and network analysis to achieve their goals. In this review, we describe these GI prediction approaches in detail, as well as their critical associated aspects, especially the reliable assessment of their predictive abilities. However, we begin with describing experimental techniques for identifying GIs, as they form the conceptual foundation and sources of data for most of the approaches described subsequently.

## Experimental Techniques for Identifying Genetic Interactions

In simple haploid organisms that can be easily manipulated via genetic engineering, such as yeast or *E. coli*, the detection of GIs can be performed by manipulating two genes at the same time, e.g., knocking them out, assessing a given phenotype, and comparing it to the result of the manipulation of single genes. Larger unbiased GI networks can be obtained using high-throughput screens. Three main high-throughput platforms exist for discovering these networks: synthetic gene array (SGA) (Tong et al., 2001), epistatic miniarray profiles (E-MAP) (Collins et al., 2010), and diploid based synthetic lethality analysis on microarrays (dSLAM) (Pan et al., 2004). In SGA, a yeast strain carrying a query mutation is crossed with an ordered array of approximately 5000 viable yeast gene deletion mutants (representing approximately 80% of all yeast genes (Baryshnikova et al., 2010)) and the meiotic progeny harboring both mutations is scored for fitness (Tong et al., 2001, 2004; Tong and Boone, 2006). To measure fitness, sizes of colonies of double and single mutants grown are measured in a high-throughput manner and single vs. double mutant fitness measurements are compared to identify GIs. In the related E-MAP approach, a rationally chosen subset of genes is studied, e.g., genes belonging to a given pathway or process, and all GIs between pairs of genes in this subset are analyzed using the SGA technique (Collins et al., 2010). In dSLAM, deletion strains containing molecular barcodes and a microarray detection technique are used to measure relative growth rates of mutant yeast strains in competition (Pan et al., 2004). Using these three approaches and other related ones, millions of gene–gene combinations have been tested in yeast, and hundreds of thousands of interactions have been discovered (Boone et al., 2007; Costanzo et al., 2010).

In human and other higher eukaryotic cells, high-throughput analysis of GIs is more difficult due to a larger number of genes and even larger number of gene pairs and higher combinations, lower efficiency of genetic engineering, and absence of resources like the yeast knockout collection, among others. Current approaches usually focus on identifying GIs involving a gene of interest, such as the p53 tumor suppressor. In such studies, isogenic wild-type (WT) cell lines and those mutant for the gene of interest are used, e.g., p53 WT vs. p53 mutant. Then, shRNA or CRISPR screens are performed to identify differentially lethal genes (Berns et al., 2004; Barbie et al., 2009; Luo et al., 2009; Scholl et al., 2009). Such screens can also be performed using small molecules that target specific proteins (Turner et al., 2008; Roller et al., 2012). A few studies have used pooled shRNA and shRNA pairs to query specific pathways for GIs in human cells (Bassik et al., 2013; Vizeacoumar et al., 2013). Because of the difficulty in accurately measuring colony size and fitness in higher order organisms, a recent set of studies have instead used a cell’s phenotype to measure the effect after siRNA-mediated knockdown (Laufer et al., 2013, 2014). After staining treated cells with markers for DNA and cell morphology, they ran automated image analysis to extract over 100 distinct descriptors. These descriptors were then combined into a single score in order to estimate the level of GI for each gene pair. These experiments have generated the largest known set of cancer-specific GIs that are expected to yield novel knowledge about cancer biology and possibly many more GIs using the methods discussed in this review.

## Characterizing and Predicting Genetic Interactions

The first high-throughput genetic interaction (GI) data sets in yeast allowed researchers to analyze the biological and topological properties of these interactions and their networks (Boucher and Jenna, 2013). Among the first of these efforts, Kelley and Ideker (2005) analyzed the correspondence between GIs and the protein interaction network in yeast and found that a substantial fraction of GIs corresponded to either within- or between-pathway interactions. Of these, they found that negative GIs (interactions that negatively impact and diminish a given phenotype), especially synthetic lethals, correspond more significantly to between-pathway interactions, as they represent cases of pathways and genes/proteins within them compensating for each others’ functions (Hartman et al., 2001). A more recent analysis of a much larger GI dataset, Bellay et al. (2011) confirmed this interpretation of negative GIs, while also indicating that positive GIs (interactions that positively impact and augment a given phenotype) are harder to interpret and may indicate both within- and between-pathway/complex interactions. Other studies have related GIs to evolutionary and functional relationships between genes (Ma et al., 2008; VanderSluis et al., 2010; Koch et al., 2012; Michaut and Bader, 2012), and we refer the reader to Boucher and Jenna (2013) for a detailed review of these results. Similar analyses have been conducted for explaining the mechanistic relevance of synthetic lethal interactions to metabolic networks (Palumbo et al., 2007). These studies established the relevance and utility of GIs for studying the functional landscape of the cell in terms of its various components, such as genes, pathways, and complexes.

### Broad Feature Engineering and Classification-Based Approaches

**Figure 1:**
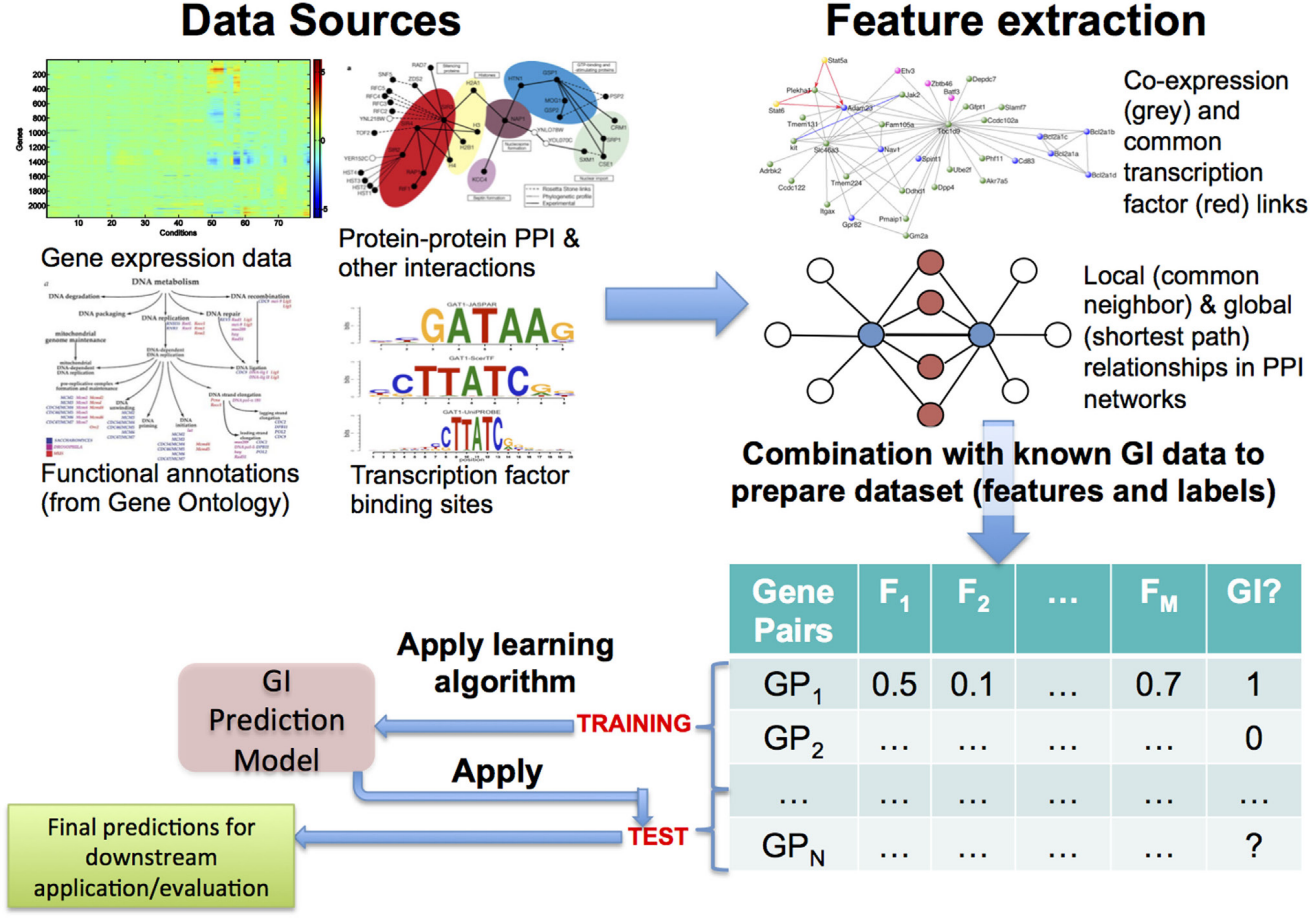
**Overview of the most commonly used approach to predicting genetic interactions (GIs)**. Here, a generally large number and variety of features are extracted from diverse data sources, examples of both of which are shown in the top panel. The feature data are combined with known GI data from public databases like BioGRID, leading to a feature + label table/matrix. Some of the gene pairs in this table, whose GI status is known, are used as training examples, from which a GI prediction model is learnt using an appropriate algorithm. Finally, the model is applied to test gene pairs to make predictions of their GI status, which can be used for downstream evaluations and/or applications.

Based on biological inferences such as those mentioned earlier, most of the early approaches to this problem followed the hypothesis that GIs and functional relationships correspond to each other well and adopted the methodology shown in Figure 1 (Tong et al., 2004; Onami and Kitano, 2006; Zhong and Sternberg, 2006; Paladugu et al., 2008; Chipman and Singh, 2009; Ulitsky et al., 2009; Pandey et al., 2010). Predictive features regarding GIs are quantified from genomic data sources, such as gene expression, protein–protein interactions (PPI), functional/pathway annotations, and evolutionary patterns. These quantifications, often a large number of them, are collected as *features* or *attributes* describing the gene pairs, and a *training set* is constructed using the available GI dataset, which is most often obtained from a public data source, such as BioGRID (Chatr-Aryamontri et al., 2015) or IntAct (Orchard et al., 2014). Finally, predictive models (Kuhn and Johnson, 2013) are learnt from this training set. To ensure that the models are actually predictive, they are rigorously evaluated using methodologies like cross-validation and metrics like precision-recall and area under the ROC curve (AUC) (Davis and Goadrich, 2006; Fawcett, 2006; Kuhn and Johnson, 2013). Finally, these models can be applied to previously unseen gene pairs to predict whether they may represent novel GIs or not.

Wong et al. (2004) presented the first successful application of this approach for *Saccharomyces cerevisiae* GIs, specifically synthetic sick and lethal (SSL) interactions. Using 123 features derived from a variety of genomic datasets and a decision tree predictor, they were able to achieve accurate predictions at a rate of 31%, two orders of magnitude higher than 0.56% success rate expected of unguided experimental screening. Zhong and Sternberg (2006) adopted a similar approach to infer GIs in *Caenorhabditis elegans* (worm). To supplement the much smaller amount of genomic data available for worm, they used orthology to transfer relevant and reliable data from fly and yeast and derive features. They used a logistic regression model to generate a predicted GI network of 18,183 interactions covering 2254 genes. Several other efforts have taken a similar feature extraction and predictive modeling approach (Paladugu et al., 2008; Chipman and Singh, 2009).

In previous work (Pandey et al., 2010), some of us developed a more comprehensive approach to GI prediction by addressing some important limitations of Wong et al. (2004)’s work. Most importantly, Wong et al. included some two-hop or transitive features for gene pairs A–B that were defined by searching if there exists a functional relationship between genes A and C and a known GI (specifically, SSL) between genes B and C. In this way, they incorporated known GI data into the features constituting the training set. While these features turned out to be substantially predictive in the cross-validation experiments on the training set, the applicability of this approach to organisms with no or very little GI data available is questionable. Thus, we designed a completely GI-independent set of 152 features derived from orthogonal genomic data sources like gene expression, PPI, functional/pathway annotations and evolutionary patterns. Table 1 lists some of the most predictive of these features, grouped by the data source they were derived from, and ranked in terms of their discriminative power. Data sources with high functional relevance, such as functional annotations, gene expression, and PPI, yielded the most discriminative features for this problem. Along with features like these, we extended the single decision tree predictor used by Wong et al. (2004) to a collection of six different predictors (*ensemble*) and employed balancing of the GI and non-GI examples in the training set to address the class imbalance issue, both of which are useful practices for biomedical prediction problems (Haibo and Garcia, 2009; Yang et al., 2010). Owing to these innovations, our system produced state of the art performance for *S. cerevisae* GI prediction (Lu et al., 2013), and was considered a major advance in computational biology (Mak, 2011).

**Table 1:** **Examples of features derived from a variety of data sources that were found to be discriminative between GI and non-GI gene pairs in our previous work on GI prediction (Pandey et al., 2010)**.

Category/data source	Feature description	KS statistic	KS *p* value
Functional information	Co-membership in the same KEGG pathway	0.4388	0
	Similarity of two genes using their annotations to GO BP terms and semantic similarity between the terms (Tao et al., 2007)	0.2306	0
	Number of functions shared by two genes [calculated here using the 138 most populated GO BP terms recommended by Myers et al. (2006)]	0.1861	0
	Similarity of two genes using their annotations to GO CC terms and semantic similarity between the terms (Tao et al., 2007)	0.1826	0
	Similarity of two genes using their annotations to GO MF terms and semantic similarity between the terms (Tao et al., 2007)	0.0763	0
Protein–protein interaction (PPI) network	Number of communities derived from PPI network that two proteins are co-members of	0.2257	0
	Length of shortest path between two	0.14	0
	Common neighborhood similarity [topological overlap (Zhang and Horvath, 2005)] between two proteins	0.0991	0
	Number of cliques in the PPI network (Zhu et al., 2008) two proteins are co-members of	0.0839	0
	Co-membership in modules discovered from PPI network (Zhang and Horvath, 2005)	0.0456	3.33E-15
	Degree of vertex corresponding to an edge in the PPI network in its edge graph version (edge degree)	0.0444	2.08E-14
	Betweenness of the edge between two proteins in the PPI network	0.0444	2.13E-14
	Presence (1)/absence (0) of an interaction between two proteins	0.0443	2.20E-14
Gene expression data (pairwise correlation of expression profiles)	From Brem et al. (2002)’s data set	0.0904	0
	From Spellman et al. (1998)’s data set	0.0594	0
	From Mnaimneh et al. (2004)’s data set	0.0471	0
	From Hughes et al. (2000)’s data set	0.0219	2.76E-04
Sequence similarity (pairwise BLAST comparison of protein sequences)	Length of alignment	0.0272	2.25E-06
	E-value of alignment	0.0271	2.30E-06
	Bit score of alignment	0.0271	2.38E-06
	Percentage identity in alignment	0.0271	2.38E-06
	Number of mismatches in alignment	0.0268	3.21E-06
	Number of gaps included in alignment	0.0235	7.00E-05
Others	Mutual information between the phylogenetic profiles of two proteins	0.0673	0
	Number of mutant phenotypes shared by two genes	0.0268	3.41E-06

*Also shown are the Kolmogorov–Smirnov test statistic scores and *p*-values indicating the relative discriminative power of these features*.

Subsequently, several studies have sought to improve this performance by enhancing the prediction algorithm used in this system, rather than focusing on adding in new features. Wu et al. (2014) converted the ensemble into a supervised approach by weighing the contributions of the individual classifiers by their respective performance (AUC score), and consequently obtained an improvement in performance. In parallel, some of us presented a systematic and rigorously evaluated framework for learning *supervised ensembles* from a large set of *heterogeneous* predictors (Whalen et al., 2015), such as those used in the above studies. Indeed, the application of this framework to GI prediction using our previously prepared dataset (Pandey et al., 2010) boosted the performance (AUC score) from 0.734 to 0.812, thus establishing a new benchmark for GI prediction. These results indicate that paying close attention to machine learning aspects, such as the use of supervised heterogeneous ensemble methods, can lead to significant improvements in our ability to address difficult biomedical prediction problems.

### Approaches Based on Evolutionary Patterns and Phenotype Data

More recently, the rapid generation and annotation of biomedical data have motivated researchers to investigate novel features for GI prediction. Lu et al. (2013) proposed an innovative approach of identifying synthetic lethal gene pairs based on evolutionary patterns. Using features derived from a phylogenetic tree, their approach identifies cases of “functionally asymmetrical” pairs of proteins A → B in a complex such that A’s function is dependent on B, but not vice versa. If they find pairs of the kind A → B and C → B in this search, but no such relationship between A and C, they hypothesize that A and C have a negative GI as A is probably compensating for C and vice versa. Due to its focus on pairs within individual protein complexes, this approach was able to achieve higher specificity but lower sensitivity in their predictions as compared to the broader feature-based studies like ours (Pandey et al., 2010). In a related study, Li et al. (2011) considered the relationship between protein domains and synthetic lethal GIs between their corresponding genes. By applying a maximum likelihood estimation approach to the currently available set of GIs in *S. cerevisiae* and the known domain structure of its proteins, they identified 3848 confident domain GIs among 1027 unique domains. Next, by matching these domains to the structures of *S. cerevisiae* proteins, they were able to confidently identify 133 synthetic lethal GIs, of which 22 were novel and potentially valid. These studies indicate the utility of a detailed study of evolutionary patterns for discovering novel GIs, and this utility will only grow with the increase in rapid sequencing and characterization of genomes and their constituent genes, proteins, and other elements (Morozova and Marra, 2008).

Another data source that has emerged recently and has been utilized in innovative approaches for GI prediction is high-throughput phenotyping (Houle et al., 2010). Using data from various phenotype ontologies (Robinson and Webber, 2014); Hoehndorf et al. (2013) assigned genes to their functions (GO terms) if a mutation in that gene is marked as causing a phenotype related to a given GO term. They were then able to predict GIs using the semantic similarity (Pesquita et al., 2009) between the GO annotations of genes. Thus, although GI prediction was only an indirect goal of this study, it laid down a path to utilize phenotype data, which are intimately connected to the definition of GIs. Calzone et al. (2015) formalized this relationship between phenotypes, loss or gain of function mutations, and GIs by statistically projecting the available data about these characteristics onto established Boolean network models of specific biological processes like the MAPK pathway. After this projection, it is possible to infer novel GIs by identifying pairs of genes involved in these processes whose combined mutational phenotypes are significantly different from their individual mutational phenotypes. To the best of our knowledge, this study represents the first instance of combining first principles-based regulatory networks with GI information to gain further knowledge about biological processes. We expect substantial progress in this direction as more -omics data are generated, and more accurate and finer granularity first principles-based models are developed using the rich biological knowledge extracted from them (Huang, 2004).

### Approaches Based (Almost) Exclusively on Existing GI Data

Finally, the availability of increasing amounts of experimental GI data, such as from SGA (Costanzo et al., 2010) and E-MAP (Collins et al., 2010) technologies, has opened several novel GI prediction avenues based on data imputation and matrix completion techniques. Qi et al. (2008) presented the first such approach, where they defined a graph diffusion kernel based on the observation that paths of odd lengths are expected to connect genes in synthetic lethal interactions in the current GI network. Cross-validation experiments on a GI network obtained from BioGRID (Chatr-Aryamontri et al., 2015) validated the predictive potential of this approach. Ryan et al. (2010) evaluated several data imputation techniques used for gene expression data (Liew et al., 2011), such as (weighted) KNNImpute and Local Least Squares (LLSImpute), for the imputation of missing, i.e., currently undiscovered, GIs in the E-MAP dataset. This evaluation showed that weighted KNNImpute and LLSImpute are the most effective for this task. Jarvinen et al. (2008) attempted to improve on these results by using the more systematic matrix approximation method for data imputation, but evaluated it on a relatively small (26 × 26) GI matrix. Importantly, these methods do not take the network structure of GI datasets into account, a limitation that was addressed in other studies. For instance, Alanis-Lobato et al. (2013) utilized several measures for the proximity of genes based on the *common neighborhood* structure of a GI network. These measures quantify the proximity between two genes/proteins using some variant of the number of common neighbors shared by them, and have been shown to be very useful for protein interaction network analysis (Pandey et al., 2014). The proximity matrix covering all the genes in the original network is then processed in a network-embedding framework (Boguna et al., 2009) to prioritize the most likely candidate GIs. Finally, Zitnik and Zupan (2015) recently developed a network guided-matrix completion (NG-MC) approach, where prior information in the form of orthogonal networks, like protein interaction networks, is used to guide the imputation process. The essence of NG-MC lies in the transfer of “information” in the form of latent features between neighboring genes/protein in the orthogonal networks to the probability of a GI between the corresponding genes. Evaluation on four different E-MAP datasets showed that NG-MC significantly outperformed other data imputation methods due to its incorporation of the orthogonal networks and their structure as prior information.

In summary, the pressing need to develop computational prediction methods for GIs and the growing availability of a variety of -omics datasets has led to the successful pursuit of a variety of innovative ideas and computational models. Using these rich data and the valuable biology learnt from them (Huang, 2004), we expect this field to grow substantially as progress is made in all these directions. With this progress, we also expect a rising ability to tackle the harder problem of discovering context-specific GIs, which we discuss in the next section.

## Discovery of Context-Specific Genetic Interactions

Genetic interactions are likely to vary significantly between different contexts. For instance, a context where a single amino acid is missing from growth media may induce fewer GIs than broadly acting perturbations such as heat-shock. Additionally, the relevant GIs will change whether one is examining a cell with a disease phenotype as opposed to a “normal” cell – this is especially the case in tumor cells. Thus, to truly understand the genetic landscape of an organism we must understand the context specificity of its GIs. In fact, a recent study in *Drosophila* showed that the majority of GIs might be context-dependent (Chari and Dworkin, 2013), but most of these contexts remain poorly studied.

As a result of this scarcity in our knowledge of context-dependent GIs, the prediction of these interactions is more difficult than normal GI prediction. To predict GIs for a specific context, such as a disease, we need a thorough understanding of the phenotypes, underlying biological networks, and genomic alterations representative of the disease – data that are often unavailable. Despite these challenges, a select number of computational methods have been successful in predicting context-specific GIs. By focusing on a specific disease, phenotype, or genes, models can be improved by incorporating mechanistic information and known interaction networks.

For instance, in the prototypical case of BRCA1 and PARP1, it is accepted that BRCA1 is involved in the repair of double-stranded DNA breaks (Roy et al., 2012), while PARP1 is involved in the repair of single-strand DNA breaks (Okano et al., 2003). Knowing that DNA replication can convert single-strand breaks to double-strand breaks, it was hypothesized that PARP1 inhibition would cause the accumulation of double-strand breaks, and thus would be particularly lethal to BRCA-deficient cells (Bryant et al., 2005; Farmer et al., 2005). van Pel et al. (2013) further exploited the genomic instability of cancer to locate relevant genetically interacting gene pairs. Since chromosome replication, maintenance, and segregation are conserved processes, they could be modeled in *S. cerevisiae*. Using experimentally validated interaction networks of genes involved in chromosomal instability, they focused on two dominating common processes – DNA replication/repair and the mitotic machinery – and identified new hub genes that were involved in these processes. Looking for GIs between cancer gene orthologs and predicted “hub” genes in these pathways, they experimentally validated GIs between cancer genes and CTF4-related proteins. Deshpande et al. (2013) presented a similar comparative genomics approach for transferring SL interactions from yeast to human, and experimentally verified the top predictions. Their results indicated that such interactions could offer therapeutic targets for cancers harboring mutations in SMARCB1 or ASPSCR1.

This knowledge-based approach has been more broadly applied to locate potential synthetic lethal pairs involved in cancer metabolism. Often, one form of an enzyme (an isozyme) is lost due to a large-scale deletion at a specific locus, but the cell is able to survive due to other isozymes of the lost enzyme acting to relieve the metabolic burden. Inhibiting the function of the other isozymes in these scenarios would lead to the complete ablation of that enzymatic process and thus would lead to cell death. This was demonstrated in glioblastoma when it was observed that inhibition of ENO2 was selectively toxic to ENO1 deficient glioblastoma cells (Muller et al., 2012). Due to the *a priori* knowledge of ENO1’s metabolic function, the authors of this study were able to prune their search of potential SL partners to isozymes catalyzing the same metabolic step. This approach was later expanded by Aksoy et al. (2014) to use TCGA (Cancer Genome Atlas Research et al., 2013) genotyping data along with curated metabolic pathways to predict SL isozyme partners across all metabolic genes and across all cancer types. They identified over 4104 candidate metabolic vulnerabilities present in 1019 tumor samples and 482 cell lines and found that up to 44% of these vulnerabilities could be targeted by at least one FDA approved drug, further emphasizing the therapeutic potential for such methods.

Other cancer-specific SL detection approaches rely on *a priori* hypotheses about the effect of SL interaction on cancer-related processes, which have been recently characterized by extensive genomic data (Zhang et al., 2011; Cancer Genome Atlas Research et al., 2013). The DAISY approach (Jerby-Arnon et al., 2014) uses three distinct inferences to detect novel SL pairs in cancer genome data:

- SL genes will have significantly lower rates of co-mutation or co-loss than non-SL gene pairs.
- The SL partners of gene A can be detected by searching for other genes whose under expression or loss induces the essentiality of A in shRNA screens.
- SL genes are involved in similar pathways and thus will be coexpressed.

The respective inverses of these inference theories were applied to also detect Synthetic Dosage Lethal (SDL) pairs. DAISY was tested against known cancer SL and SDL pairs, achieving an AUC value of 0.779, was used to predict and validate novel SL partners of the VHL tumor suppressor gene, and was then used to create genome-wide cancer-specific SL (2816 interactions covering 2077 genes) and SDL (3635 interactions covering 3158 genes) networks. Additionally, using TCGA data from specific cancer types, Jerby-Arnon et al. (2014) used DAISY to create cancer-specific SL and SDL networks populated with SL and SDL interactions that they predict to be specific for a given cancer type. Their results further show how much methods and networks can be used in the precision medicine setting for determining successful drug treatments or prognosis. Lu et al. (2015) used a similar hypothesis – when one member of an SL partner is lost, the other tends not to be lost – to examine cancer genome and gene expression patterns to predict genetically interacting gene pairs. Their model achieved an AUC of 0.75 when tested against empirically measured cancer GIs and created a genome-wide list of SL interactions covering up to 591,000 gene pairs. Though they pooled data from a variety of different cancer types, such an approach could be adapted to use genome evolution data from a specific cancer or disease type to predict context-specific SL pairs. Thus, in a manner similar to the analysis/prediction of other interactions networks (Huang, 2004), approaches such as these reveal how the consideration of network or mechanistic information can allow for the prediction of specific GIs that might be overlooked by more general methods.

## Evaluation of GI Predictions

The evaluation of predictions made by the approaches discussed in this article, i.e., how many of them are (in)correct, is perhaps the most complicated aspect of GI prediction. Since this aspect has not been studied systematically for this problem, we discuss below its critical components as analyzed in the prediction of other interactions/networks, such as PPI (Skrabanek et al., 2008), genetic regulatory networks (De Smet and Marchal, 2010), and drug–target interaction networks (Kuhn et al., 2008). We will emphasize the critical basics of these components as they apply to GI prediction. For in-depth details of these basics, we refer the reader to other excellent reviews (Schrynemackers et al., 2013) and data mining texts (Tan et al., 2005).

### Benchmark Datasets Representing “Ground Truth”

The first requirement for evaluating any kind of prediction (GIs in our case) is a collection of examples with true labels (here, whether it represents a GI or not). The predictions made for the examples in this collection, commonly known as a benchmark dataset, can be matched against their true labels to assess the predictive ability of the algorithm being evaluated. However, since no such sizeable benchmarks have been curated for GIs, the studies discussed above have curated their own datasets to evaluate their algorithms. Although several studies have made their best efforts to evaluate and compare performance across multiple datasets, these results still may not be comprehensive due to incompleteness and biases within these datasets. Thus, representative benchmark datasets, such as those that have been created (simulated) for genetic regulatory networks (Cantone et al., 2009; Pinna et al., 2011; Schaffter et al., 2011), are a pressing necessity for the field of GI prediction. We are confident that as more large-scale GI datasets, such as Costanzo et al. (2010)’s, are generated and the need for (standardizing) GI prediction is established, such benchmarks will become more readily available.

Another exciting opportunity for benchmarking has been offered by the rapid growth of crowdsourcing-based efforts, such as DREAM challenges (Stolovitzky et al., 2007; Jarchum and Jones, 2015), to solve biomedical problems such as network inference (Marbach et al., 2012; Meyer et al., 2014) and protein function prediction (Pena-Castillo et al., 2008; Radivojac et al., 2013). Here, appropriate datasets pertinent to the target problem are released to the community, with an aim to develop effective solutions by leveraging the “wisdom of crowds.” Most importantly, the evaluation of the solutions submitted to these challenges is carried out in a comprehensive completely transparent manner on an independent test set. Thus, for GI prediction, such challenges can offer a reliable evaluation mechanism. Again, with the generation of large-scale GI datasets, we are ever closer to the organization of such challenges.

### Evaluation Methodology

Despite the benefits, an independent dataset is often not available for evaluation in biomedical settings. In such settings, simulating a test set from the available training set itself is the best option for reliable evaluation, and *cross-validation* (Schrynemackers et al., 2013) (CV) is the most commonly used methodology for performing this simulation. In *k*-*fold* CV, the training dataset is randomly split into *k* equally sized subsets, referred to as folds. In each CV round (*split*), *k* − 1 of these subsets are used for training, and the resultant model is applied to generate predictions for the remaining subset. Table 2 enumerates this process for *k* = *n*, the number of examples in the training set [known as leave-one-out cross-validation (LOOCV)]. Repeating this process over all *k* folds generates predictions for the whole training set, which can then be matched against the true labels of the examples to quantify the performance of the algorithm being evaluated. For GI prediction, the predominantly assumed form of examples used for training or prediction here is gene pairs.

**Table 2:** **Visual depiction of the *k*-fold cross-validation approach, where *k* = *n* (leave one out) cross-validation (LOOCV) procedure applied to a data set with *n* examples**.

CV round	Training examples	Test example
1	2, 3, 4, 5, 6, …, *n*	1
2	1, 3, 4, 5, 6, …, *n*	2
3	1, 2, 4, 5, 6, …, *n*	3
.	.	.
.	.	.
.	.	.
*n*	1, 2, 3, 4, 5, …, *n* − 1	*n*

*In each round, one of the examples is reserved for testing the predictive model learnt over the remaining training examples. Other forms of *k*-fold CV (*k* < *n*) also operate similarly, with the difference that the training and test example splits are generated randomly from the original data set*.

While standard CV has often been effectively used in GI prediction, some serious problems arise due to the nature of GIs and the structure of the networks (Barabasi and Oltvai, 2004). Most notably, biological networks follow a skewed degree distribution, with very few of the nodes (*hubs*) having much higher degrees (more connections) than a vast majority of the nodes. Schrynemackers et al. (2013) showed that it is possible in such a network to obtain better than random interaction prediction by simply connecting any node in the test split with the more connected nodes in the training split. This result, which does not even involve the node or interaction features, emphasizes the importance of taking the network structure into account for evaluation, and presented this result as a baseline for every dataset and algorithm. Petri et al. (2015) studied this problem in the context of genetic regulatory networks, and proposed a randomized permutation-based evaluation measure to help standardize evaluation results.

Park and Marcotte (2012) investigated a different but related complexity that arises due to the (partial) mismatch between training and test splits when using CV for evaluating PPI predictions. As detailed by Figure 1 in their article, the following three categories of examples arise in a training-test split in CV:

- C1: both proteins in the test pair are covered in the training set.
- C2: one of the proteins in the test pair is covered in the training set.
- C3: none of the proteins in the test pair are covered in the training set.

Now, by evaluating seven representative algorithms in a CV setting on a standard PPI dataset, the author showed that the performance of these algorithms was significantly different for these categories. As would be intuitively expected, the performance over C1 examples was vastly better than over C2 and C3 ones, with the latter being the hardest to predict accurately. The overall performance is dominated by C1, which provides an inaccurate estimate of how the prediction algorithm will perform for examples not covered well by the training set. This is an important concern for GI prediction, as the known GIs for most organisms and conditions/contexts form a small set of the possible interactions, thus making the ability to predict such “unseen” examples accurately critical. Hamp and Rost (2015) highlighted a further complexity of this issue by demonstrating that the disparity of performance persists if the definition of “overlap” for determining C1–C3 is based on (sequence) “similarity” of proteins instead of an exact match. These results highlight the importance of reporting results individually for these categories in addition to the overall performance.

In summary, issues, such as the effect of skewed degree distribution and varying overlaps between training and test examples, should be carefully considered when evaluating GI prediction results, especially in a cross-validation setting.

### Evaluation Measures

The final component of the evaluation task is the identification of appropriate evaluation *measures* or *metrics* that can quantify how correct are the test set predictions made by an algorithm. *Accuracy*, the most straightforward measure, can be defined simply as the ratio of the number of correct predictions to the size of the test set (original set in the case of CV). However, this measure is misleading in cases like GI prediction, where the positive examples (true GIs) are a very small minority of the full dataset. As a result, a naïve algorithm that predicts every example as negative can achieve a very high accuracy, an obviously unreliable result. Measures like the *ROC curve* and the area under it (*AUC* or *AUROC* score) (Fawcett, 2006), as well as the *precision*-*recall*-*F-measure* trio and the associated area under the *precision-recall curve* (*AUPRC* score) (Davis and Goadrich, 2006) are much more reliable in such scenarios, and thus are more commonly used in GI and other interaction prediction tasks. Both these measures allow the examination of the relationship between correct and incorrect predictions, measured as the true and false positive rates in the ROC curve and precision and recall in the P–R curve, over a range of thresholds that can be applied to the scores/probabilities output by a prediction algorithm. However, precision and recall, and hence, F-measure, are calculated for the negative and positive classes individually, while the ROC curve and its AUC score are the same regardless of which of the classes is named positive and which negative. Due to this, the precision-recall-F-measure trio and the precision-recall curve are more suited for evaluating the prediction ability of the generally significantly smaller positive class of GIs, as compared to non-GIs. This class-specific characteristic also enables easy assessment over multiple classes, such as positive, negative and non-GIs.

In summary, reliable assessment of a GI prediction algorithm can be done effectively by make appropriate choices of the benchmark dataset(s), evaluation methodology and evaluation metrics. However, we would like to point out some GI-specific aspects of evaluation that should also be considered:

### Multi-Class Nature of GIs

Genetic interactions, by their definition, can be both positive and negative. Thus, ideal GI prediction systems should be able to predict both these classes of GIs, in addition to identifying which gene pairs represent non-GIs. Most of the GI prediction approaches so far have been restricted to predicting only synthetic (sick and) lethal [S(S)L] GIs, primarily due to the lack of sufficient data on other types of GIs. Now, with the generation of large datasets, we expect that more comprehensive GI prediction algorithms will be developed, and they will be robustly evaluated using class-specific measures such as precision recall F- measure.

### Network-Based and Orthogonal Evaluation

We discussed earlier that GI networks, such as other biomedical networks, have well-defined structural properties (Barabasi and Oltvai, 2004). One such biologically important property is *modularity* (Kaltenbach and Stelling, 2012). This property indicates that nodes and interactions in these networks are organized into functionally coherent *modules*, which interact through linker biomolecules to collectively perform higher cellular functions. Thus, in addition to evaluating individual interaction predictions, one should also consider evaluating the modularity of the network resulting from the predicted GIs (Costanzo et al., 2010; Bellay et al., 2011) and how it compares with the training/original GI network. This evaluation can also be strengthened by assessing the functional enrichment of the modules in terms of functional annotations like those provided by Gene Ontology and canonical pathways (Huang da et al., 2009), and comparing with the enrichment of the original/training network. Note that in such enrichment comparisons, it is not only necessary to assess if one is better than the other but also if the predictions uncover reliable biomedical knowledge. This is one of the end goals of effective GI prediction.

### Need for More Rigorous Evaluation Methodologies

Some complications when using standard methodologies like cross-validation (CV) for GI prediction evaluation were discussed above. Now, with the generation of larger GI datasets, and the rapid development and application of more sophisticated prediction methodologies, like ensemble learning (Yang et al., 2010) and deep learning (LeCun et al., 2015), new complications might arise. One of these is the possibility of *data leakage* and *overfitting* (Tarca et al., 2007; Kaufman et al., 2011) between training and test splits, during which the selection/evaluation of the most predictive model might be biased due to an inappropriate consideration of the test set labels. This problem usually arises due to multiple rounds of learning from the given training set/split, such as the selection of the most predictive features, and subsequent learning of a predictive model based on those features. Indeed this problem has been witnessed in early work on identifying predictive biomarkers of disease from (generally small) gene expression datasets (Ambroise and McLachlan, 2002; Saeys et al., 2007). One of the ways to address this problem is to separate the training set into many parts, one each for each operation that involves the use of the training labels. This can be further systematized by using a nested cross-validation procedure to make more comprehensive use of the training set. This approach was used in our previous work on GI prediction with much success (Whalen et al., 2015).

To conclude, the rigorous evaluation of GI prediction algorithms is a critical but complicated task. However, we believe that this task can be reliably accomplished based on the findings and guidelines laid out in this section.

## Discussion

Our understanding of the network of GIs is likely in its infancy, especially in complex, multi-cellular organisms. However, as has been witnessed for other types of interactions (Huang, 2004), we are at the cusp of inferring useful and actionable biology from the existing and soon-to-come GI networks. A subset of these interactions, synthetic lethal interactions, is poised to provide a rich source of therapeutically exploitable vulnerabilities for precision medicine. Since it is unlikely that the space of possible interactions, especially context-dependent ones, can ever be explored systematically using experimental approaches, there is a great need for computational methods that predict GIs. In the future, we expect that machine learning approaches trained on gold standard GIs sets will be combined with patient-acquired data, e.g., RNA-seq and whole-exome/genome, to predict candidate interactions. These interactions may then be tested using induced pluripotent stem cells (iPSC), organoids or patient-derived xenografts (PDX). In this regard, it will be important to perform more systematic exploration of GIs in selected contexts to create gold standards for computational methods to be trained. The increasing applicability of CRISPR technology may make synthetic lethality screening broadly possible, using not just a single guide but combinations of two or more RNA guides. Extending these methods to more than pairs of genes (triplets etc.) represents a major computational and statistical challenge and will likely require even more sophisticated computational methodologies.

## Author Contributions

NSM, OE, and GP drafted, revised, and finalized the manuscript.

## Conflict of Interest Statement

The authors declare that the research was conducted in the absence of any commercial or financial relationships that could be construed as a potential conflict of interest.
